# The COOH-terminal domain of huntingtin interacts with RhoGEF kalirin and modulates cell survival

**DOI:** 10.1038/s41598-018-26255-1

**Published:** 2018-05-22

**Authors:** Hollis McClory, Xiaolong Wang, Ellen Sapp, Leah W. Gatune, Maria Iuliano, Chiu-Yi Wu, Gina Nathwani, Kimberly B. Kegel-Gleason, Marian DiFiglia, Xueyi Li

**Affiliations:** 1 Laboratory of Cellular Neurobiology and Department of Neurology, Massachusetts General Hospital and Harvard Medical School, Charlestown, MA 02129 USA; 2 School of Pharmacy, Shanghai Jiao Tong University, Minhang District, Shanghai, China

## Abstract

Human huntingtin (Htt) contains 3144 amino acids and has an expanded polyglutamine region near the NH_2_-terminus in patients with Huntington’s disease. While numerous binding partners have been identified to NH_2_-terminal Htt, fewer proteins are known to interact with C-terminal domains of Htt. Here we report that kalirin, a Rac1 activator, is a binding partner to C-terminal Htt. Kalirin and Htt co-precipitated from mouse brain endosomes and co-localized at puncta in NRK and immortalized striatal cells and primary cortical neurons. We mapped the interaction domains to kalirin674-1272 and Htt2568-3144 and determined that the interaction between kalirin and Htt was independent of HAP1, a known interactor for Htt and kalirin. Kalirin precipitated with mutant Htt was more abundant than with wild-type Htt and had a reduced capacity to activate Rac1 when mutant Htt was present. Expression of Htt2568-3144 caused cytotoxicity, partially rescued by co-expressing kalirin674-1272 but not other regions of kalirin. Our study suggests that the interaction of kalirin with the C-terminal region of Htt influences the function of kalirin and modulates the cytotoxicity induced by C-terminal Htt.

## Introduction

Huntington’s disease (HD) is a dominantly inherited neurodegenerative disorder characterized by progressive uncontrolled choreiform movements, cognitive decline and personality disturbance. The genetic mutation of HD causes an elongation of a tract of glutamines near the NH_2_-terminus of huntingtin (Htt), a ubiquitously expressed and brain-enriched protein comprising 3144 amino acids^1^. Although the exact pathogenic mechanism of HD is not fully understood, the elongated polyglutamine (polyQ) tract in Htt has been found to cause defects in multiple cellular pathways^2^, which eventually lead to cell death. Studies suggest that polyQ expansion alters the conformation of huntingtin^3–5^, thereby subjecting it to increased proteolysis and impaired clearance of the N-terminal regions and/or altered protein interactions.

Numerous proteins interact with huntingtin at the N-terminus^6,7^. The function of huntingtin has been linked mainly to interactions at its NH_2_ domain and to the toxicity associated with polyQ expansion. Some binding partners that have been identified to interact within the C-terminal region of huntingtin suggest roles associated with membrane functions (dynamin, Atgs) and cell toxicity (NF-κB)^8–10^. Strikingly, recent findings suggest that the C-terminus of huntingtin can mediate toxicity when expressed *in vitro* and *in vivo*^8,9^.

In this study we characterized kalirin, a guanine nucleotide exchange factor (GEF) for Rac1^11^, as an interactor for the C-terminal domain of Htt. Kalirin was altered in association with mutant Htt and in activating Rac1 when binding to mutant Htt. Expression of a C-terminal domain of Htt (Htt2568-3144) caused cellular toxicity, which was reduced by co-expressing a kalirin domain that interacts with Htt. Our findings suggest that the Htt-kalirin interaction may modify the function of kalirin and cellular toxicity.

## Results

### Kalirin interacts with huntingtin at membranes

**Figure 1:**
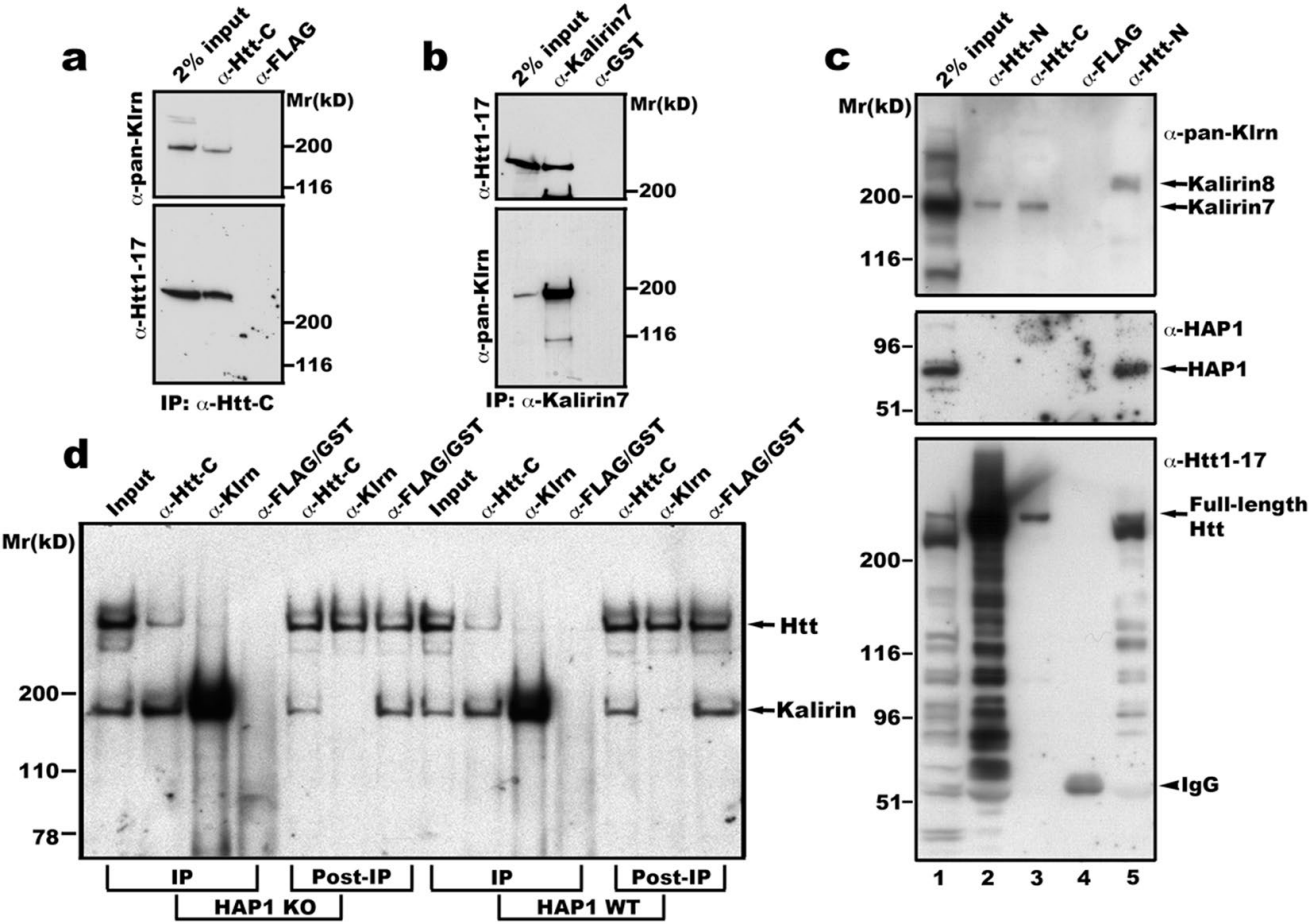
Kalirin interacts with Htt in brain membranes. (**a**) and (**b**) Kalirin and Htt are mutually co-precipitated. Mouse brain membranes as described in Method were solubilized in lysis buffer containing 1% Triton X-100 and centrifuged to discard unsolubilized membranes. The supernatant was incubated with antibodies against Htt-C (aa2703-2911 of Htt)^36^, (**a**) or aa1641-1654 of kalirin-7 (**b**). After washes, immunoprecipitates were eluted into sample buffer and analyzed by SDS-PAGE and Western blot with indicated antibodies. Shown are blot analyses from one of four experiments for both (**a**) and (**b**). (**c**) and (**d**) Interaction between Htt and kalirin is independent of HAP1. Triton X-100 solubilized mouse brain endosomes (lanes 1, 2, and 3) or mouse embryonic stem cell total membranes (lane-5) were used for immunoprecipitation with antibodies against aa181-810 of Htt (Htt-N, lanes 2 and 5) or aa2703-2911 of Htt (Htt-C, lane-3) or the FLAG tag (lane-4). For IgG control precipitation, solubilized endosomes and ES cell membranes were mixed and incubated together with anti-FLAG antibodies (lane-4). Precipitates were washed, eluted into SDS-PAGE sample buffer and analyzed by Western blot with antibodies against *pan*-kalirin, HAP1, or aa1-17 of Htt. The arrowhead labeled with IgG heavy chain indicates the 55 kD band present in the complex precipitated by anti-FLAG antibodies. We noticed that the majority of Htt proteins were cleaved during experimental procedures (Input, lane-1). This was not a surprise because it is well known that NH_2_ Htt is prone to cleavage by proteases. This may be the reason why the antibody against Htt1-17 detected much weaker signals of full-length Htt in precipitates obtained with anti-Htt-C antibodies. (**d**) Triton X-100 solubilized cortical total membranes from HAP1 knockout (KO) and corresponding WT mouse embryos were incubated with antibodies specific for aa2703-2911 of Htt or kalirin7 or FLAG/GST (IgG controls). Precipitates were washed and analyzed by SDS-PAGE and Western blot with antibodies for detecting Htt and kalirin. Blots were first probed with anti-pan-kalirin to detect precipitated kalirin and then re-probed with anti-Htt1-17 to detect precipitated Htt.

In previous studies, we showed that optineurin and Htt are associated with inactive Rab11 and others demonstrated that optineurin, named as FIP-2, interacts with Htt^12,13^. We used association with inactive Rab11 to search for other potential Htt interacting proteins. By mass spectrometry, we identified RhoGEF kalirin from proteins precipitated by inactive GST-Rab11 (data not shown). To know if kalirin and Htt, like optineurin and Htt, interacted with each other, we performed immunoprecipitation studies using mouse brain Rab11-enriched membranes (we referred to these membrane fractions as endosomes) as sources of endogenous Htt and endogenous kalirin. The neuronal isoform of kalirin (kalirin-7) was precipitated with antibodies against amino acids 2703–2911 in Htt (Fig. 1a). Under the same conditions, kalirin-7 was not precipitated with the control antibody against the FLAG tag. Full-length Htt was also co-precipitated with antibodies against amino acids 1641–1654 of kalirin-7, but not with a control antibody against GST (Fig. 1b). The reciprocal co-precipitation of kalirin with Htt or Htt with kalirin under native conditions supports an interaction between Htt and kalirin in brain cells.

The *kalirin* gene encodes a 350 kD protein that contains two RhoGEF domains. Alternative splicing generates different isoforms with one GEF domain (kalirin-7 and kalirin-8) or two GEF domains (kalirin-9 and kalirin-12)^14^. Kalirin-8, which is expressed in non-neuronal cells, and kalirin-7, which is specific for neurons, have the same GEF domain^11,15^. Kalirin-7 has a C-terminal tail (AA1634-1654) that is absent in kalirin-8, whereas kalirin-8 has 263 more residues at the C-terminus than kalirin-7^11^. Kalirin, which is also known as duo, interacts with huntingtin-associated protein 1 (HAP1) and HAP1 associates with the NH_2_ region of Htt^16,17^.

In agreement with the association of kalirin with Rab11 (data not shown), kalirin was detected in brain endosomes (fraction-6 in Figure S1). In addition, Htt functions at Rab11-positive endosomes^12,18,19^. Therefore, we conducted immunoprecipitation studies with brain endosomes to examine if HAP1 mediated the interaction of Htt with kalirin. Antibodies against two different epitopes in Htt were used: one within aa181–810 of Htt (anti-Htt-N), the other within aa2701-2911 of Htt (anti-Htt-C). Western blot analysis with antibodies against aa1-17 of Htt showed that both anti-Htt-N and anti-Htt-C antibodies precipitated full-length Htt (lower panel, Fig. 1c). We noticed that Htt was cleaved into numerous fragments containing the NH_2_-terminus during the preparation of brain endosomal membranes. This was not a surprise because Htt is known to be sensitive to proteases^20^. Under these conditions, endogenous kalirin-7 was precipitated from brain endosomes by both Htt-specific antibodies, but not by a control antibody against the FLAG tag (Fig. 1c upper panel, lanes 2 and 3). However, neither of the antibodies directed to N- or C- terminal regions in Htt could precipitate HAP1 from mouse brain endosomal membranes **(**Fig. 1c middle panel, lanes 2 and 3**)**. On the other hand, HAP1 and kalirin-8 were co-precipitated with Htt from Triton  X-100 solubilized membranes prepared from mouse embryonic stem cells (Fig. 1c lane-5). These data suggest that the interaction of Htt with kalirin is independent of HAP1.

To provide additional evidence, we performed co-immunoprecipitation using total membranes prepared from cerebral cortices of HAP1 knockout (KO) and corresponding WT mouse embryos, which were generously provided by Xiao-Jiang Li and colleagues^21^. HAP1 immunoprecipitates were undetectable in cortices of WT or KO mouse embryos when compared to the expression levels in adult mouse brain tissues (Figure S2a). We found that anti-Htt antibodies could not efficiently enrich Htt containing the first 17aa (Fig. 1d), possibly because proteolytic cleavage in the NH_2_ region of Htt might occur during membrane preparation and/or eluting immunoprecipitates for analysis. Our data in Fig. 1d showed that kalirin was co-precipitated with Htt from HAP1 deficient mouse cortical membranes. Collectively, our data support that the interaction of Htt with kalirin on endosomal membranes does not require HAP1.

### Kalirin interacts with a C-terminal domain of huntingtin

**Figure 2:**
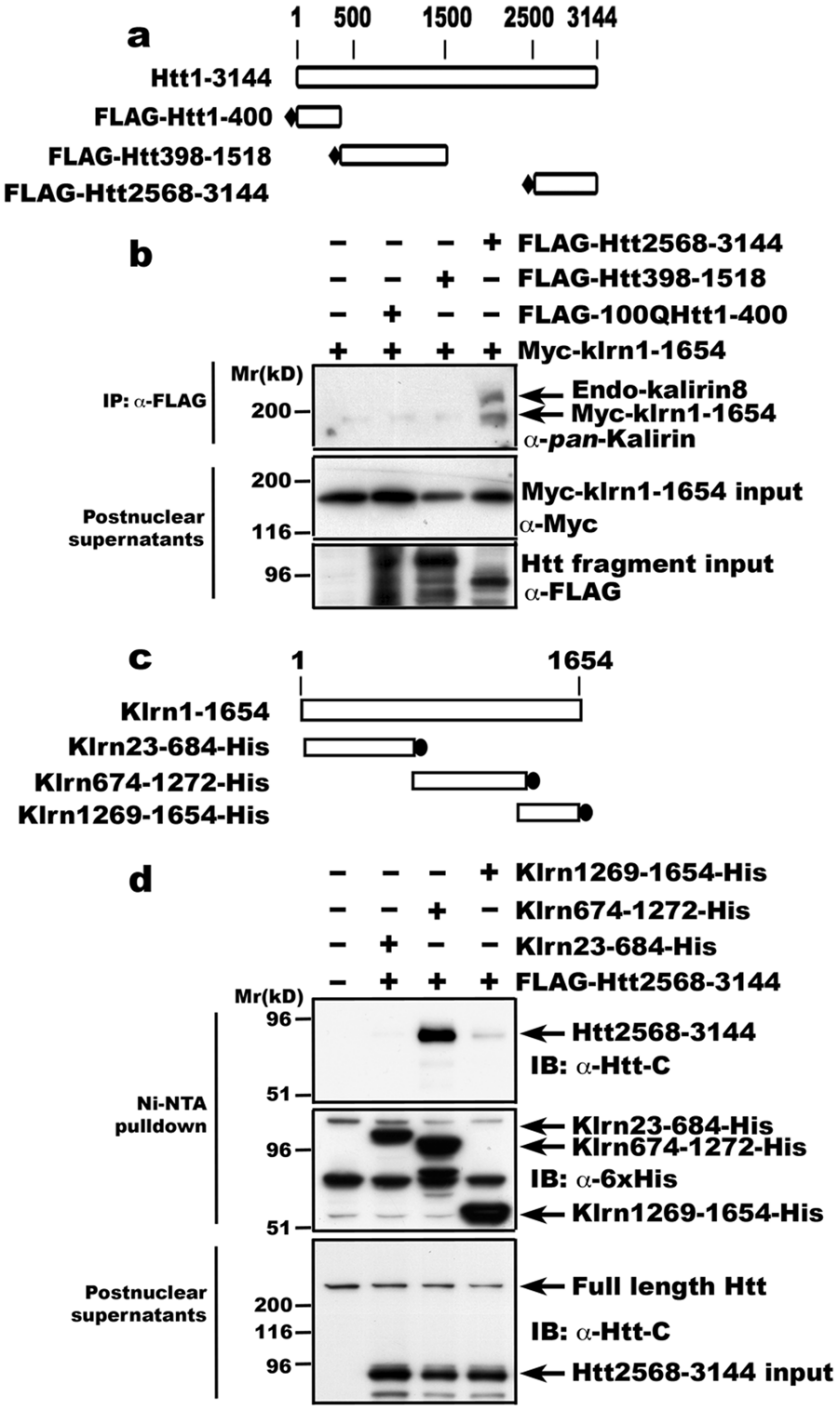
Mapping of the corresponding domains mediating the interaction between Htt and kalirin. (**a**) Diagram of the constructs expressing FLAG-tagged Htt fragments used for co-expression with His-myc-kalirin-7 in MCF-7 cells. The number of amino acids is shown above. (**b**) The C-terminal fragment of Htt interacts with kalirin. Different regions of Htt tagged with a FLAG sequence were co-expressed with 6xHis-myc-kalirin in MCF-7 cells. Immunoprecipitates obtained using anti-FLAG antibody (upper panel) and the corresponding cell lysates (middle and lower panels) were analyzed by SDS-PAGE and Western blot with anti-*pan*-kalirin antibody (upper panel), anti-myc antibody (middle panel) and anti-FLAG antibody (lower panel), respectively. Of note, the input cell lysates analyzed with anti-FLAG antibodies were run on a separate gel and 100QHtt1-400 migrated as smears possibly due to aggregate formation. Data shown are from one of three experiments. (**c**) Schematic representation of constructs expressing different regions of kalirin-7. The amino acid sequence for generating the constructs was based on rat kalirin-7^11^. The number of amino acids is shown above. (**d**) The middle fragment of kalirin-7 (Klrn674-1272) interacts with Htt. Different regions of kalirin-7 were co-expressed with FLAG-tagged Htt2568-3144 in 293 T cells and enriched using Ni-NTA resins. Proteins on Ni-NTA resins were eluted into sample buffer and analyzed by SDS-PAGE and Western blot with an antibody against aa2703-2911 of Htt. Cell lysates for lower blots were run different gels and show the presence of transiently expressed huntingtin fragment in post nuclear supernatant used for the enrichment with Ni-NTA. Shown are data from one of three experiments.

We next determined the corresponding regions in Htt and in kalirin mediating their interaction using tagged proteins expressed in MCF-7 cells, which express the non-neuronal isoform kalirin-8. His-myc-tagged kalirin-7 was co-expressed with different regions of Htt fused to a FLAG tag (Fig. 2a). We generated the fragments of Htt based on the availability of restriction endonuclease sites to facilitate the cloning and the clusters of HEAT repeats^22^. Antibodies for FLAG precipitated all of the 3 Htt fragments from cell lysates (Figure S2b). However, exogenous His-myc tagged kalirin-7 was co-precipitated only with the C-terminal domain of Htt (Htt2568-3144), but not with an NH_2_-terminal domain (Htt1-400) with expanded polyQ (100Q) or an internal domain (Htt398-1512) **(**Fig. 2b**)**. This finding of kalirin interacting with C-terminal Htt adds further evidence supporting that the interaction between Htt and kalirin is not mediated by HAP1, which is associated with NH_2_-terminal Htt^17^. Endogenous kalirin-8 was also co-precipitated with Htt2568-3144 (Fig. 2b), suggesting that both neuronal kalirin-7 and non-neuronal kalirin-8 interact with a C-terminal region within aa2568-3144 of Htt.

To look for the region in kalirin to which Htt binds, FLAG tagged Htt2568-3144 was co-expressed with different fragments of kalirin-7 (Fig. 2c). The NH_2_-terminal domain of kalirin containing the sec4 domain and the first 4 spectrin repeats was similar to the endogenously expressed kalirin-4, whereas the COOH-terminal domain harbored the single GEF domain of kalirin-7 and the internal fragment contained the spectrin repeats 5 to 9. Co-immunoprecipitation studies revealed that Htt2568-3144 interacted with an internal region of kalirin (Klrn674-1272) (Fig. 2d, third lane). The association of Htt with Klrn674-1272, which is present in both neuronal kalirin-7 and non-neuronal kalirin-8, further supports that kalirin and Htt interact with each other in both neurons and non-neuronal cells. Despite being to a much lesser degree, Htt2568-3144 was also co-precipitated with Klrn1269-1654 **(**Fig. 2d, fourth lane), which is the known GEF domain of kalirin-7/8. It is not clear whether the interaction between Klrn1269-1654 and Htt2568-3144 is direct or mediated by a substrate GTPase of kalirin-7/8.

**Figure 3:**
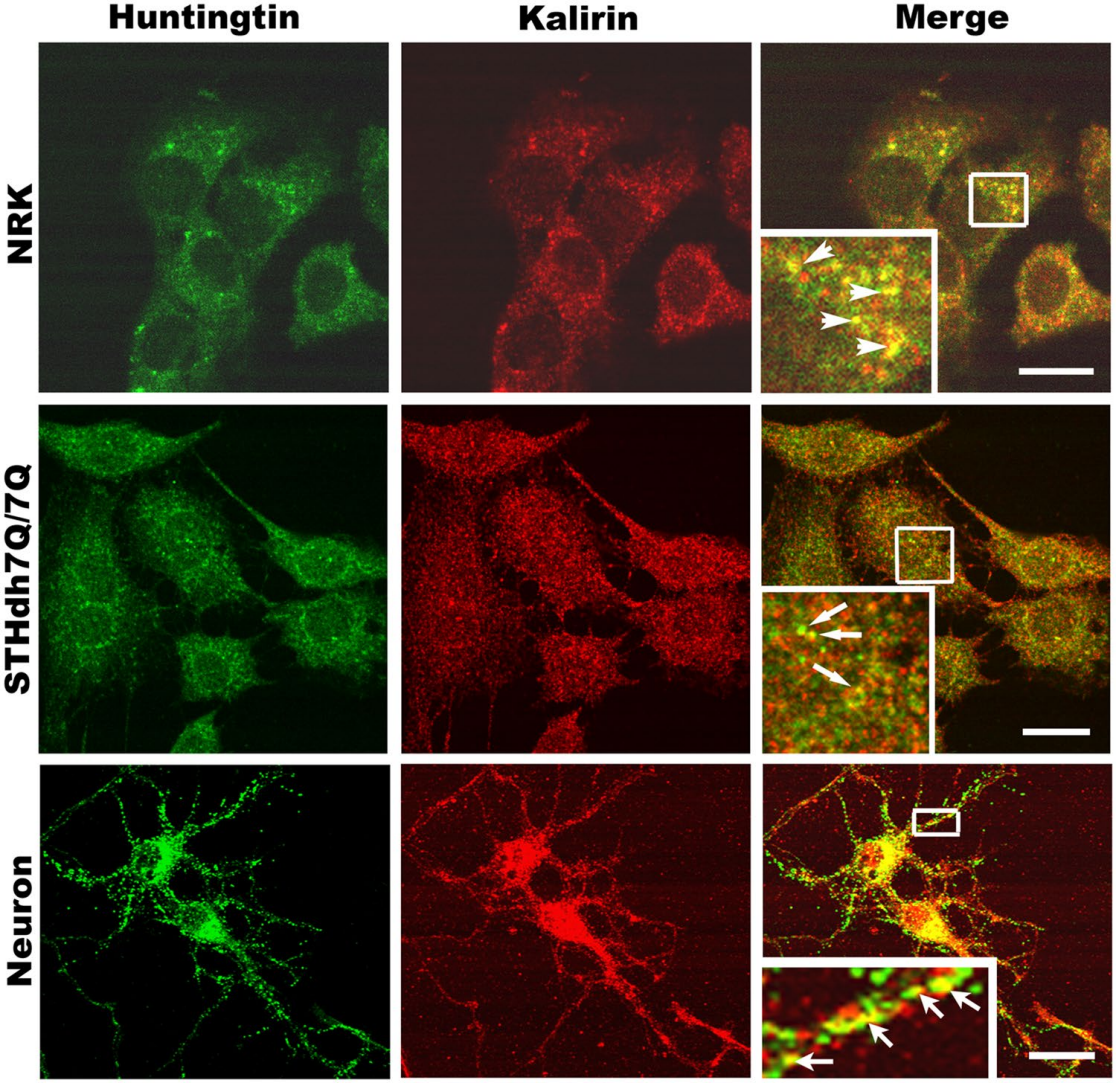
Localization of Htt to kalirin-positive structures in NRK cells, immortalized striatal cells, and primary cortical neurons. NRK cells (upper panels) or STHdh7Q/7Q (middle panels) or primary cortical neurons cultured for 9 days *in vitro* (lower panels) on glass coverslips were processed for labeling with antibodies against Htt (green) and *pan*-kalirin (red). The merged image shows areas of co-localization of Htt and kalirin in yellow. Shown are confocal images. The boxed regions over the cells in the merged photo column are enlarged at the left lower corner. Here arrows indicate structures co-labeled by antibodies against Htt and kalirin (yellow). Scale bar in the upper and middle panels is 25 μm, whereas the scale bar in the lower panel is 50 μm.

To determine if Htt and kalirin co-localize in cells we performed double immunofluorescent labeling. In NRK cells, endogenous kalirin-8 and endogenous Htt co-localized at punctate structures (Fig. 3). In primary mouse cortical neurons, immunoreactivity for both kalirin and Htt occurred in the same subcellular foci, particularly within spine laden neurites, where kalirin is known to be located^11,15^ (Fig. 3). Analysis of co-localization using JACoP, NIH imageJ^23^ revealed that endogenous kalirin and endogenous Htt highly co-localized at punctate structures in neurons and NRK cells (Pearson’s correlation co-efficient: 0.724 ± 0.07, n = 21 neuronal processes; 0.736 ± 0.07, n = 15 NRK cells). Structures co-labeled with Htt and kalirin were also observed in immortalized STHdh7Q/7Q cells (Fig. 3). Together, these data suggest that kalirin and Htt co-distribute in non-neuronal and neuronal cells.

### Interaction with mutant huntingtin alters the activity of kalirin on Rac1

**Figure 4:**
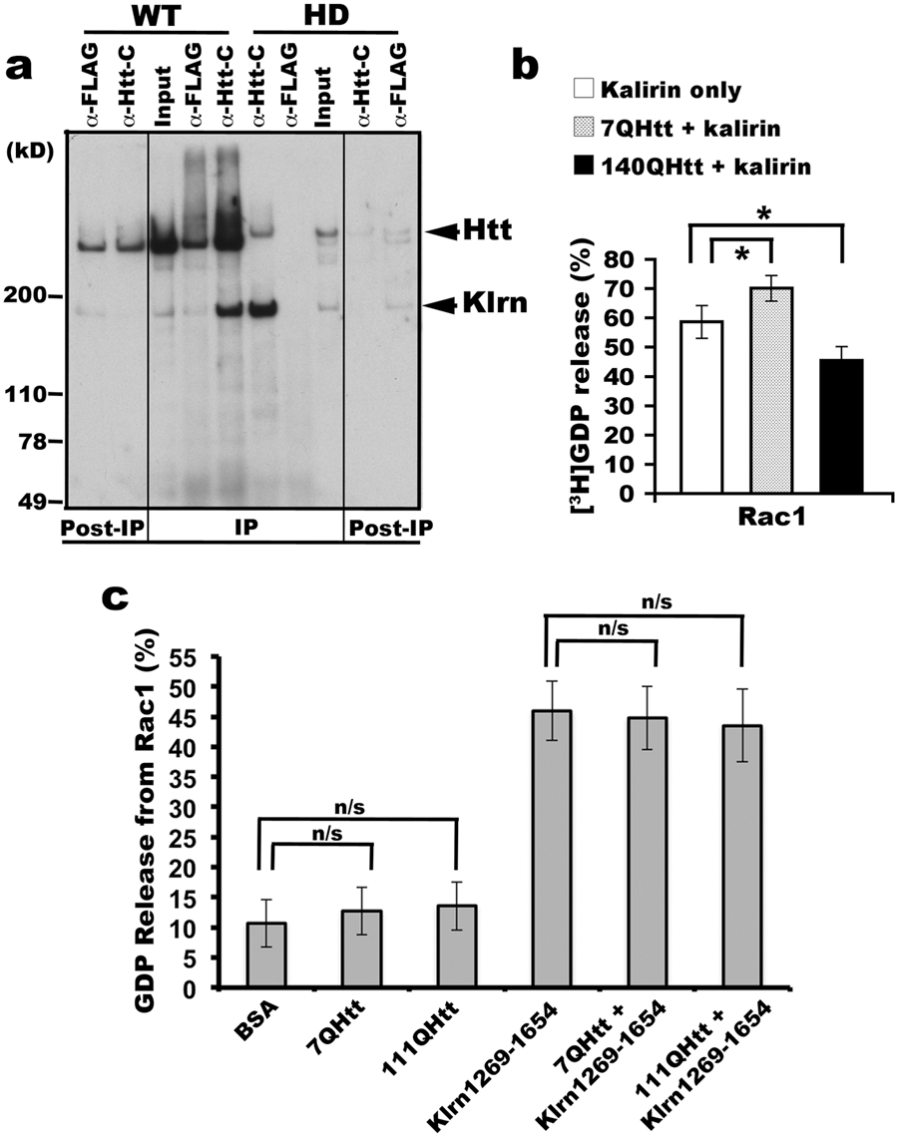
Mutant Htt compromises kalirin-enriched proteins from promoting nucleotide exchange on Rac1. (**a**) Stronger binding of kalirin to mutant Htt than to WT Htt. Triton X-100 solubilized brain endosomes prepared from WT (7Q) or HD140Q/140Q mouse brains were incubated with protein-G beads coupled with antibodies against aa2703-2911 of Htt. Precipitates were washed and analyzed by SDS-PAGE and Western blot with indicated antibodies. Of note, the detection of Htt signal in the lane identified as WT anti-FLAG was due to the leak-over of the WT anti-Htt-C sample from the adjacent lane. Shown is one blot analysis out of three experiments. (**b**) Kalirin-7 immunoprecipitated from mouse brain membranes was incubated with Htt precipitated from mouse brain cytosol (wild-type or HD140Q/140Q) at 4 **°**C for 2 hrs and used for catalyzing [^3^H]GDP release from GST-Rac1. Data are represented as mean percentage of released [^3^H]GDP from GST-Rac1 (n = 3, Mean ± SD, Student t-test: *p < 0.05). (**c**) No effect of Htt on the activity of the GEF domain of kalirin-7/8 (kalirin7aa1269-1654) in accelerating nucleotide exchange on Rac1. Htt immunoisolated with antibodies against Httaa2703-2911 from cytosol of STHdh7Q/7Q and STHdh111Q/111Q striatal cells, respectively, was incubated with kalirin7aa1269-1654 purified from bacteria and then used for catalyzing GDP release from GST-Rac1. Data are represented as mean percentage of released GDP from GST-Rac1 (n = 3, Mean ± SD, Student t-test, n/s: not significant).

Most Htt binding partners identified so far exhibit a stronger binding to mutant Htt than to wild-type Htt^6,7,24,25^. We therefore investigated if an abnormal interaction also existed between kalirin and mutant Htt. To exclude the effect of Htt abundance in WT and HD140Q/140Q mouse brain endosomes on the amount of kalirin co-precipitated with Htt, we conducted immunopreciptation with an excess of endosomal fractions to saturate the binding capacity of antibodies used for precipitating Htt. Antibodies against Htt2703-2911 robustly precipitated kalirin from both WT and HD140Q/140Q mouse brain membranes (Fig. 4a). We observed an increase in signals of kalirin co-precipitated with Htt from HD140Q/140Q mouse brain membranes relative to signals of kalirin precipitated from WT mouse brain membranes (Fig. 4a), suggesting an aberrant binding of Htt to kalirin in HD conditions.

To test if abnormal interaction with mutant Htt alters kalirin to accelerate GDP-to-GTP exchange on its substrate Rac1, we immuno-isolated kalirin from WT mouse brain endosomal membranes and Htt from WT (7Q) and HD140Q/140Q mouse brain cytosol for use in a GDP release assay. Our data in Fig. 4b shows that the catalytic activity of kalirin toward Rac1 was significantly enhanced in the presence of Htt precipitates from WT mouse brain cytosol (7Q) and reduced in the presence of Htt precipitated from HD140Q/140Q mouse brain cytosol (140Q), suggesting that the function of kalirin for activating Rac1 could be compromised in HD brain. Neither WT (STHdh7Q) nor mutant (STHdh111Q) Htt precipitated from cytosol had GEF activity toward Rac1 or affected the kalirin-7/8 GEF domain (Klrn1269-1654) in promoting nucleotide exchange on Rac1 (Fig. 4c). These data suggest that the disturbance of kalirin activity on Rac1 by mutant Htt requires the presence of the Htt-interacting domain (Klrn674-1272).

### Kalirin modulates the cytotoxicity of huntingtin C-terminal domain

**Figure 5:**
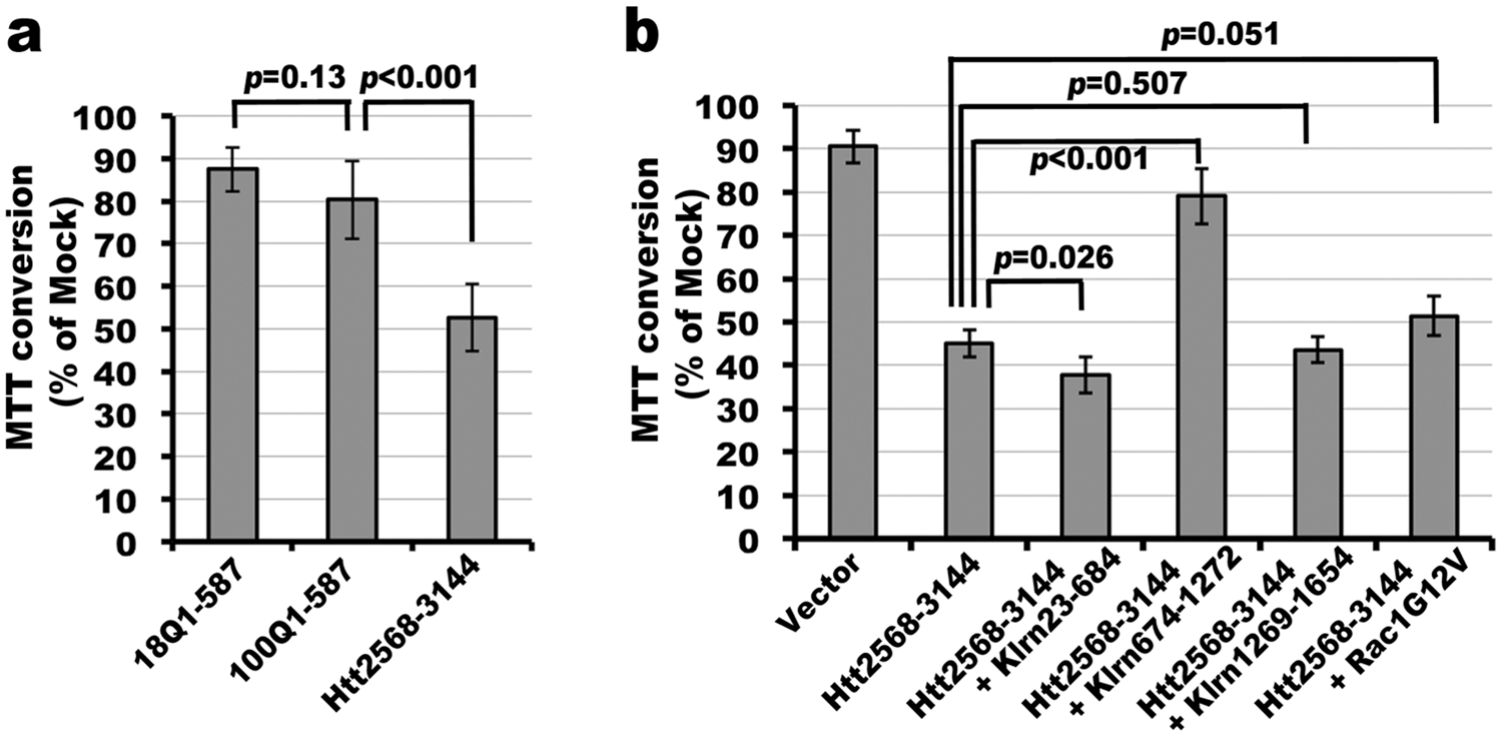
Cytotoxicity of Htt C-terminal fragment is partially attenuated upon the expression of a Htt-interacting domain in kalirin. (**a**) STHdh7Q/7Q striatal cells were transfected with plasmid DNAs expressing FLAG-tagged 18QHtt1-587, 100QHtt1-587, or Htt2568-3144 for 14 hours and processed for the MTT conversion assay. For each transfection condition, 3 independent transfections were performed. (**b**) STHdh7Q/7Q striatal cells were transfected with the Htt2568-3144 expressing plasmid with or without plasmids expressing 6xHis-tagged Klrn23-684 or Klrn674-1272 or Klrn1269-1654 or Rac1G12V for 12 to 14 hours. Appropriate amounts of empty vector DNAs were supplemented to make the amount of plasmid DNAs equal for each transfection condition. Two independent transfections were conducted for each condition. In both (**a**) and (**b**), empty pcDNA3 vector was used as the mock transfection control. Quantification of MTT conversion for each transfection condition was conducted in duplicate. The average of MTT conversion from different transfections for each condition was calculated. Data were represented as the percentage of MTT conversion in cells transfected with empty vector. Statistical significance was determined by two-tailed Student *t*-test.

We noticed that the majority of STHdh7Q/7Q cells were released into cell culture media upon transfection with the construct expressing a C-terminal domain of Htt (Htt2568-3144), which is a sign of death. To provide quantitative data, we conducted the 3-(4,5-dimethyl-2-thiazolyl)-2,5-diphenyl-2H-tetrazolium bromide (MTT) conversion assay to compare the effects of the expression of different Htt fragments. Since levels of MTT conversion into formazan were proportional to the number of viable cells, a decrease in the MTT conversion was used as a criterion of cellular toxicity. We noticed a 47% reduction in the conversion of MTT into formazan upon the expression of Htt2568-3144 compared to cells transfected with an equal amount of empty vector, indicative of increased toxicity (Fig. 5). Levels of MTT conversion in cells expressing Htt2568-3144 were significantly lower signifying less viability than those in cells expressing an NH_2_-terminal fragment (Htt1-587) of comparable size even with an elongated polyQ (100Q) tract (Fig. 5a). These data showed that the C-terminal domain of Htt is toxic to cells. Relative to empty vector, both 18QHtt1-587 and 100QHtt1-587 were toxic to cells. However, the cytotoxicity of 100QHtt1-587 was only slightly greater but did not reach statistical significance relative to the cytotoxicity of 18QHtt1-587 (Fig. 5a). Similarly, full-length mutant Htt (46Q) did not evoke significant cytotoxicity when compared to full-length WT Htt (Figure S3). This could be because the duration of the expression of mutant Htt including 100QHtt1-587 was not long enough to evoke pronounced cytotoxicity.

Since Htt2568-3144 interacted with kalirin, we examined if co-expression of the kalirin domain that interacted with Htt2568-3144 could affect the toxicity rendered by the expression of Htt2568-3144. Indeed, the MTT conversion was significantly increased in cells co-expressing Klrn674-1272 and Htt2568-3144, indicative of improved survival (Fig. 5b). However, the co-expression of the kalirin fragments that did not interact or weakly interacted with Htt2568-3144 could not ameliorate the cytotoxicity caused by overexpressing Htt2568-3144 (Fig. 5b). Expression of Klrn674-1272 alone did not significantly affect MTT conversion when compared with the expression of full-length kalirin-7 (Figure S3). Since kalirin is a GEF for Rac1, we then examined if the cytotoxicity of Htt2568-3144 might result from impaired activation of Rac1 in cells. If so, co-expression of a dominant active Rac1 mutant would attenuate the cytotoxicity rendered by Htt2568-3144. Expression of dominant active Rac1G12V mutant had no effect on MTT conversion when compared to cells transfected with empty vector (Figure S3). We observed that the co-expression of Rac1G12V led to a slight increase (14%) in the MTT conversion in cells expressing Htt2568-3144 (Fig. 5b). The observation that dominant active Rac1 was unable to mitigate the cytotoxicity of Htt2568-3144 suggests the involvement of other kalirin substrate(s) or interacting partner(s). Future studies are needed to find such substrate(s) or interacting partner(s).

## Discussion

In contrast to the numerous binding partners identified to interact with the NH_2_-terminal domain in Htt, which has an expanded polyglutamine tract in HD, few interactors have been described to the C-terminal domain in Htt^6,7^. Here we identify an interaction between kalirin, a Rho GEF, and a C-terminal region of Htt. An interaction between Htt and kalirin (named as HAPIP by the authors) was previously suggested based on a large screen of Htt interaction partners using high throughput yeast two-hybrid and affinity pull down assays^26^. Kalirin, also known as duo, is an interaction partner of HAP1^16^. We mapped the interaction domain in Htt and the domain in kalirin. The region in kalirin that we found to interact with a C-terminal fragment of Htt is also known to interact with HAP1^16^. HAP1 interacts with an NH_2_-terminal domain (aa1-230 with 44Q) in Htt and an internal segment (kalirin7aa656-1242) in kalirin^16,17^. Three lines of evidence suggest that HAP1 was not required for the interaction of Htt2568-3144 with kalirin on endosomal membranes. First, HAP1 and kalirin are associated with different Htt domains; HAP1 interacts with N-terminal Htt, whereas kalirin is associated with C-terminal Htt. Second, both Htt-specific antibodies used in our experiments precipitated kalirin but not HAP1 from brain endosomes. Lastly, kalirin was co-precipitated with Htt from cortical total membranes of mouse embryos that were deficient in HAP1. The functional relevance of these associations is still not clear; one possibility is that HAP1 and Htt normally compete for binding to kalirin to engage different functions.

The functional significance of the association of kalirin, a Rho GEF, with inactive Rab11 remains to be defined. Coordinated operation of Rab mediated membrane traffic with Rho governed cytoskeleton remodeling occurs in many cellular events. For example, the budding of transport carriers involves the integration of Rab engaged assembly of vesicle machinery and Rho involved polymerization of actin networks at budding sites of donor compartments^27^. In the nervous system, LTP induction involves activity-dependent growth of dendritic spines, which needs extra membranes containing AMPA receptors (functional plasticity) and actin cytoskeleton reorganization (structural plasticity). The mechanisms that underlie the coordination between Rab and Rho GTPases are poorly understood. The association of kalirin (a RhoGEF) with Rab11 may provide a bridge for the coordination between Rab11 and Rho.

The interaction between Htt and kalirin may be highly dynamic. The increased binding of mutant Htt to kalirin possibly impairs the disassembly of the Htt-kalirin complex, thereby leading to more kalirin co-precipitated with mutant Htt. PolyQ expansion in Htt may also alter the conformation of the Htt-kalirin complex and thereby interfere with kalirin dependent activation of Rac1. Rac1 has a critical function in remodeling the actin cytoskeleton during several important cellular events including growth of dendritic spines for LTP induction^15,28,29^. Signs of impaired actin polymerization are seen in STHdhQ111 striatal cells as well as in hippocampal slices from HD mice^30,31^. Thus, the abnormal function of kalirin in regulating Rac1 in the presence of mutant Htt may contribute to some of the impaired actin remodeling seen in the context of HD.

Consistent with a recent report by El-Daher and colleagues^8^, we observed cytotoxicity when a C-terminal fragment of Htt (aa2568-3144) was expressed. To our surprise, the toxicity rendered by Htt2568-3144 was significantly greater than the cytotoxicity of polyQ-containing NH_2_-terminal fragment with an expanded polyQ tract (100QHtt1-587). The cytotoxicity evoked by the C-terminal fragment of Htt was attenuated upon co-expression of a domain to which Htt bound in kalirin. The functional significance of these findings remains speculative. It is noteworthy that Mende-Mueller *et al*. showed the overproduction of C-terminal fragments of Htt in HD brains compared to control brains^32^. The authors noticed that levels of two low molecular weight (42 kD and 48 kD) C-terminal fragments were elevated in HD brains by 20- and 5- fold, respectively^32^.

In summary, we show that endogenous Htt and kalirin interact and co-localize at neuronal membranes. The domain in Htt important for the interaction with kalirin is located at the C-terminus of Htt. The GEF activity of kalirin on Rac1 is impaired by its interaction with mutant Htt. Overexpressing Htt2568-3144 caused cellular toxicity, which could be reduced by co-expressing the region in kalirin that interacts with Htt. Our findings suggest that the Htt-kalirin interaction may be involved in modulating Rac1 activity and cellular toxicity.

## Materials and Methods

### Animals

Homozygous Q140 knock-in HD (HD^140Q/140Q^) and wild-type mice were maintained and bred at the Animal Core Facility of the Massachusetts General Hospital. Experiments involved in the use of mice were performed according to the institutional and US National Institute of Health guidelines and approved by the Massachusetts General Hospital Subcommittee on Research Animal Care.

### Plasmid DNA

The cDNA encoding mouse Rac1 (BC051053.1) was amplified by reverse transcriptase polymerase chain reaction and cloned into the BamHI and XhoI sites of pGEX-6p. Dominant active Rac1 mutant (Rac1G12V) was generated by site-specific mutagenesis and subcloned into the BamHI and XhoI sites pcDNA3-HA. To generate the plasmid expressing kalirin7aa23-684 and kalirin7aa674-1272, the corresponding DNA fragment was amplified from the pEAK-kalirin7 plasmid (a generous gift of Dr. Betty A Eipper at the University of Connecticut Health Center, USA) by PCR using primer pairs: 5′ cgcgaattcggatcttttcggaatgatg and 5′ tatacccgggctgctggaactgcttgat for kalirin7aa23-684 and 5′ cggtcaattggatgcggtccaggaactga and 5′ tatacccgggctttcgagctgacttccgt for kalirin7aa674-1272. PCR products were treated with EcoR I/Xma I for kalirin7aa23-684 and Mfe I/Xma I for kalirin7aa674-1272, respectively. The DNA fragment encoding kalirin7aa1269-1654 was obtained by digesting pEAK-kalirin-7 with EcoR I and Not I. These kalirin-7 DNA fragments were cloned into the EcoR I and Xma I sites of pCMV-SPORT6 with an HA tag at the NH_2_-terminus and a 6xHis tag at the C-terminus of recombinant proteins. The DNA fragment encoding kalirin7aa1269-1654 was also cloned into the EcoRI and NotI sites of pGEX-6p. The indicated regions of Htt were amplified by PCR and cloned into FLAG-tagged pcDNA_3_ plasmid. All constructs were verified by DNA sequencing.

### GST fused protein expression and purification

BL21 cells were induced to express GST-Rac1 for 4 hr, harvested, and homogenized in cold lysis buffer (0.1 M phosphate, 0.3 M NaCl, 10% glycerol, protease inhibitors) by sonication. Crude homogenates were centrifuged at 4 °C 14,000 rpm for 45 min to remove cell debris and unbroken cells. The resulting supernatants were incubated with glutathione beads. After extensive washes, GST-Rac1 or GST-kalirinaa1269-1654 bound onto glutathione beads were eluted and dialyzed against 20 mM Tris/Cl (pH7.4) and 5 mM EDTA (pH7.1) to remove nucleotides bound on Rac1. Purified GST-Rac1 proteins were divided into aliquots, frozen in liquid nitrogen, and stored at −80 °C until use.

### Preparation of total cellular membranes, brain endosomes, and cytosol

We used hypertonic homogenate buffer (50 mM HEPES-Na, pH7.0, 200 mM NaCl, 5 mM MgCl_2_, 1 mM DTT, 1 mM EDTA, 0.25 M sucrose and protease inhibitors) to minimize the chance of the oligomerization of Htt. Fresh mouse brain tissues were collected and minced into pieces. Brain pieces in homogenate buffer were passed through a dounce homogenizer for 12–15 strokes on ice. For membrane preparation from cultured mouse embryonic stem cells, cells were harvested and lysed in cold homogenate buffer by passing through a 27 1/2-gauge needle for 15–20 strokes. Homogenates were centrifuged at 4 °C 14,000 rpm for 10 min and the pellet (P1) was discarded. The resulting supernatant (S1) was either directly centrifuged at 4 °C 100,000 × g for 1 hr to prepare S2 (referred to as cytosol) and P2 (referred to as total cellular membranes) or used for the preparation of endosomal membranes (see below).

Brain endosomal membranes were prepared as described previously^33^. In brief, the above S1 supernatant (approximately 4 ml) was supplemented with sucrose to a final concentration of 40% (v/v), overlaid with a linear sucrose gradient, and subjected to a centrifugation at 4 °C 100,000 × g for 24 hr. After centrifugation, 12 fractions, each in 1 ml, were collected from the top of the gradients and analyzed by Western blot to detect the enrichment of Rab11. The fraction enriched in Rab11 was referred to as brain endosomes (typically fractions 5, 6 and/or 7).

### Protein precipitation

For immunoprecipitation of endogenous Htt and/or kalirin, Triton-solubilized brain endosomal membranes were incubated with antibodies specific for Htt2703-2911 (MAB2051, Serotec) or Htt181-810 (MAB2166, EMD Millipore) or Klrn1641-1654 (Everest Biotech) or FLAG (M2, Sigma-Aldrich) or GST (GE Healthcare). Protein complexes were captured onto protein-G beads pre-equilibrated with the sucrose-free homogenate buffer containing 1% Triton X-100 by incubating at 4 °C for 2 hours with gently mixing and eluted into SDS-PAGE sample buffers for SDS-PAGE and Western blot analysis.

For mapping the interaction domains in Htt and kalirin, MCF7 cells were transiently transfected with corresponding plasmids using X-tremeGene 9 reagents, harvested, re-suspended in lysis buffer containing 1% Triton-X 100, and incubated on ice for 30 min with gentle mixing every 5 min. Homogenates were centrifuged at 4 °C 12,000 rpm for 10 min. The resulting supernatants were incubated with antibodies specific for FLAG pre-bound onto protein-G beads or Ni-NTA beads. Proteins on beads were washed and eluted into SDS-PAGE sample buffers for SDS-PAGE and Western blot analysis.

### Guanine nucleotide exchange assay

[^3^H]GDP release and subsequent quantification were performed as previously described^12,18,19^. In brief, 0.5 μg of GST-Rac1 was immobilized on glutathione beads and loaded with 20 pmol of [^3^H]GDP (11.9 Ci/mmol, Amersham) in loading buffer (20 mM HEPES, pH7.2, 20 mM potassium acetate, 1 mM DTT, 5 mM EDTA) at 30 °C for 30 min. After loading, free [^3^H]GDP was removed by washing glutathione beads twice in ice-cold loading buffer containing 10 mM MgCl_2_. Kalirin-Htt complexes were diluted in 50 μl of assay buffer (20 mM HEPES, pH7.2, 5 mM Mg(OAc)_2_, 1 mM DTT and 0.75 mM GTP/GDP) and added to the [^3^H]GDP-loaded GST-Rac1. The nucleotide exchange reaction was initiated by incubating at 30 °C. After the exchange reaction was stopped, GST-Rac1 on beads was collected by a centrifugation and washed twice in cold buffer (20 mM Tris/Cl, pH7.4, 20 mM NaCl, 5 mM MgCl_2_ and 1 mM DTT) for scintillation counting, thus only the remaining [^3^H]GDP on GST-Rac1 was counted. Data were represented as mean percentage of released [^3^H]GDP (subtracting the counting of the remaining [^3^H]GDP on GST-Rac1 from total binding or no GEF control).

### Cell culture

Media and supplements were purchased from Invitrogen Inc. and fetal calf serum was obtained from Atlanta Biologicals. MCF7 and NRK cells were cultured in DMEM media supplemented with 10% fetal calf serum, L-glutamine and penicillin/streptomycin at 37 °C in a cell culture incubator (Thermo Electron Corporation). STHdh7Q/7Q striatal cells were obtained from Coriell Institute for Biomedical Research and were cultured in complete DMEM media at 33 °C. Primary mouse cortical neurons were prepared and cultured as described previously^34^.

### SDS-PAGE and Western blot

SDS-PAGE and Western blot analysis were performed as described previously^12^. Concentrations of primary antisera were as follow: Ab1 polyclonal antibody specific for aa1-17 of Htt (1 μg ml^−1^), polyclonal anti-pan kalirin (1:750; Millipore), MAB2051 monoclonal anti-Htt2703-2911 (Htt-C, 1:1,000; Serotec), polyclonal anti-HAP1 (1:50; Martin *et al*.^35^), monoclonal anti-myc (1:1,000; Covance), M2 monoclonal anti-FLAG (1:5,000; Sigma-Aldrich), and monoclonal anti-6xHis (1:5,000; EMD Biosciences). Peroxidase-conjugated secondary antibodies (Jackson Laboratories) were diluted 1:5,000. For probing with different primary antibodies, the blots were either cut into strips or stripped with stripping buffer after the detection of one protein was complete. The blots were developed using enhanced ECL (Pierce).

### Blot image digitalization

Films were scanned using the Epson Perfection V750 Pro scanner and the digitalized images were stored and used for densitometry analysis using NIH ImageJ where applied. For the presentation in figures, digitalized blot images were processed and cropped using PhotoShop to show the interested protein signals. Full-length gels/images for the preparation of Figs 1a–c and 2 are included as Supplementary information. Blots in supplementary Figure S1 were cut into strips for probing with different primary antibodies.

### Immunofluorescence microscopy

Immunofluorescence analysis was performed as standard procedures. In brief, NRK cells or primary mouse cortical neurons at DIV9 on glass coverslips were washed twice in PBS, fixed in 4% paraformaldehyde/PBS at room temperature for 15 min, incubated with 50 mM NH_4_Cl to quench free aldehyde groups, washed twice in PBS and labeled with primary antibodies against pan-kalirin (polyclonal against the 4 to 7 spectrin repeats of kalirin; EMD Millipore) and Htt (MAB2166). Targeted proteins were visualized by incubation of cells on glass coverslips with secondary antibodies conjugated with BODIPY or Cy-3 (Jackson Laboratory). Microscopy was performed using a 60x or 100x oil Nikon Plan Apo objective lens mounted on an inverted Nikon Eclipse TE300 fluorescent microscope. Images were collected using a Bio-Rad Laser-sharp confocal system equipped with krypton-argon and blue diode lasers, and merged using PhotoShop.

### Determination of cell viability

Cell viability was determined by the MTT assay. STHdh7Q/7Q striatal cells in 24-well plates were transiently transfected with indicated plasmids using X-tremeGene 9 (Roche) for 14 hr. After culture, cells were changed into fresh complete media containing 0.5 mg ml^−1^ of MTT (Sigma) for 15 min at 37 °C. After the incubation, the media were carefully removed and 250 μl 100% DMSO was added to each well to dissolve formazan blue. Two aliquots of dissolved formazan from each well of the 24-well plate, each in 100 μl, were transferred into a 96-well plate for measurement at absorbance 540 nm on a multi-well spectrophotometer. Higher absorbance at 540 nm indicates more viable cells and less cytotoxicity.

## Electronic supplementary material

Supplementary figures and whole blots

## References

[1] A novel gene containing a trinucleotide repeat that is expanded and unstable on Huntington’s disease chromosomes. The Huntington’s Disease Collaborative Research Group. *Cell* **72**, 971–983 (1993). 10.1016/0092-8674(93)90585-e 8458085

[2] Jimenez-Sanchez, M., Licitra, F., Underwood, B. R. & Rubinsztein, D. C. Huntington’s Disease: Mechanisms of Pathogenesis and Therapeutic Strategies. *Cold Spring Harb Perspect Med* (2016). 10.1101/cshperspect.a024240 PMC5495055 27940602

[3] Cui X (2014). TR-FRET assays of Huntingtin protein fragments reveal temperature and polyQ length-dependent conformational changes. Sci Rep.

[4] Fodale V (2014). Polyglutamine- and temperature-dependent conformational rigidity in mutant huntingtin revealed by immunoassays and circular dichroism spectroscopy. PLoS One.

[5] Vijayvargia R (2016). Huntingtin’s spherical solenoid structure enables polyglutamine tract-dependent modulation of its structure and function. Elife.

[6] Harjes P (2003). & Wanker, E. E. The hunt for huntingtin function: interaction partners tell many different stories. Trends Biochem Sci.

[7] Li SH, Li XJ (2004). Huntingtin-protein interactions and the pathogenesis of Huntington’s disease. Trends Genet.

[8] El-Daher MT (2015). Huntingtin proteolysis releases non-polyQ fragments that cause toxicity through dynamin 1 dysregulation. Embo J.

[9] Ochaba J (2014). Potential function for the Huntingtin protein as a scaffold for selective autophagy. Proc Natl Acad Sci USA.

[10] Takano H, Gusella JF (2002). The predominantly HEAT-like motif structure of huntingtin and its association and coincident nuclear entry with dorsal, an NF-kB/Rel/dorsal family transcription factor. BMC Neurosci.

[11] Penzes P (2000). An isoform of kalirin, a brain-specific GDP/GTP exchange factor, is enriched in the postsynaptic density fraction. J Biol Chem.

[12] Li X (2008). A function of huntingtin in guanine nucleotide exchange on Rab11. Neuroreport.

[13] Hattula K, Peranen J (2000). FIP-2, a coiled-coil protein, links Huntingtin to Rab8 and modulates cellular morphogenesis. Curr Biol.

[14] Rabiner CA, Mains RE, Eipper BA (2005). Kalirin: a dual Rho guanine nucleotide exchange factor that is so much more than the sum of its many parts. Neuroscientist.

[15] Penzes P (2001). The neuronal Rho-GEF Kalirin-7 interacts with PDZ domain-containing proteins and regulates dendritic morphogenesis. Neuron.

[16] Colomer V (1997). Huntingtin-associated protein 1 (HAP1) binds to a Trio-like polypeptide, with a rac1 guanine nucleotide exchange factor domain. Hum Mol Genet.

[17] Li XJ (1995). A huntingtin-associated protein enriched in brain with implications for pathology. Nature.

[18] Li X (2009). Disruption of Rab11 activity in a knock-in mouse model of Huntington’s disease. Neurobiol Dis.

[19] Li X (2009). Mutant huntingtin impairs vesicle formation from recycling endosomes by interfering with Rab11 activity. Mol Cell Biol.

[20] Saudou F, Humbert S (2016). The Biology of Huntingtin. Neuron.

[21] Li SH (2003). Lack of huntingtin-associated protein-1 causes neuronal death resembling hypothalamic degeneration in Huntington’s disease. J Neurosci.

[22] Palidwor GA (2009). Detection of alpha-rod protein repeats using a neural network and application to huntingtin. PLoS Comput Biol.

[23] Bolte S, Cordelieres FP (2006). A guided tour into subcellular colocalization analysis in light microscopy. J Microsc.

[24] Ratovitski T (2012). Huntingtin protein interactions altered by polyglutamine expansion as determined by quantitative proteomic analysis. Cell Cycle.

[25] Zuccato C, Valenza M, Cattaneo E (2010). Molecular mechanisms and potential therapeutical targets in Huntington’s disease. Physiol Rev.

[26] Kaltenbach LS (2007). Huntingtin interacting proteins are genetic modifiers of neurodegeneration. PLoS Genet.

[27] Anitei M, Hoflack B (2012). Bridging membrane and cytoskeleton dynamics in the secretory and endocytic pathways. Nat Cell Biol.

[28] Hall A (1998). Rho GTPases and the actin cytoskeleton. Science.

[29] Xie Z (2007). Kalirin-7 controls activity-dependent structural and functional plasticity of dendritic spines. Neuron.

[30] Lynch G (2007). Brain-derived neurotrophic factor restores synaptic plasticity in a knock-in mouse model of Huntington’s disease. J Neurosci.

[31] Munsie L (2011). Mutant huntingtin causes defective actin remodeling during stress: defining a new role for transglutaminase 2 in neurodegenerative disease. Hum Mol Genet.

[32] Mende-Mueller LM, Toneff T, Hwang SR, Chesselet MF, Hook VY (2001). Tissue-specific proteolysis of Huntingtin (htt) in human brain: evidence of enhanced levels of N- and C-terminal htt fragments in Huntington’s disease striatum. J Neurosci.

[33] McClory H (2014). Glucose transporter 3 is a rab11-dependent trafficking cargo and its transport to the cell surface is reduced in neurons of CAG140 Huntington’s disease mice. Acta Neuropathol Commun.

[34] Li X (2010). Aberrant Rab11-dependent trafficking of the neuronal glutamate transporter EAAC1 causes oxidative stress and cell death in Huntington’s disease. J Neurosci.

[35] Martin EJ (1999). Analysis of Huntingtin-associated protein 1 in mouse brain and immortalized striatal neurons. The Journal of comparative neurology.

[36] Wilkinson FL (1999). Localization of rabbit huntingtin using a new panel of monoclonal antibodies. Brain Res Mol Brain Res.

