# Supplementary material for: The COOH-terminal domain of huntingtin interacts with RhoGEF kalirin and modulates cell survival
Source: Sci Rep. 2018 May 22;8:8000. doi: 10.1038/s41598-018-26255-1 (PMC5964228; doi:10.1038/s41598-018-26255-1)
Supplement: Supplementary file 1 — Supplementary figures and whole blots [file 41598_2018_26255_MOESM1_ESM.pdf]

Supplementary information includes supplementary figures and full-length blots for Figures 1 and 2.

The COOH-terminal domain of huntingtin interacts with RhoGEF kalirin and modulates cell survival

Hollis McClory[1, #], Xiaolong Wang[2, #], Ellen Sapp[1], Leah Htt Gatune[1], Maria Iuliano[1], Chiu-Yi Wu[1], Gina Nathwani[1], Kimberly B Kegel-Gleason[1], Marian DiFiglia[1], Xueyi Li[1, 2, *]

Supplementary figure S1. Mouse brain endosomes were prepared as described previously (1). Equal volume of each indicated fraction collected from the top to bottom was analyzed by SDS-PAGE and Western blot with indicated antibodies. Shown are data from one of at least 6 gradient preparations. Fractions 9-12 (which were equivalent to the loaded post-nuclear supernatants before centrifugation) represent cytosolic fractions. The arrow on the top of the photograph indicates the Rab11-enriched fraction (herein fraction-6) that we referred to as endosomes and used for IPs.

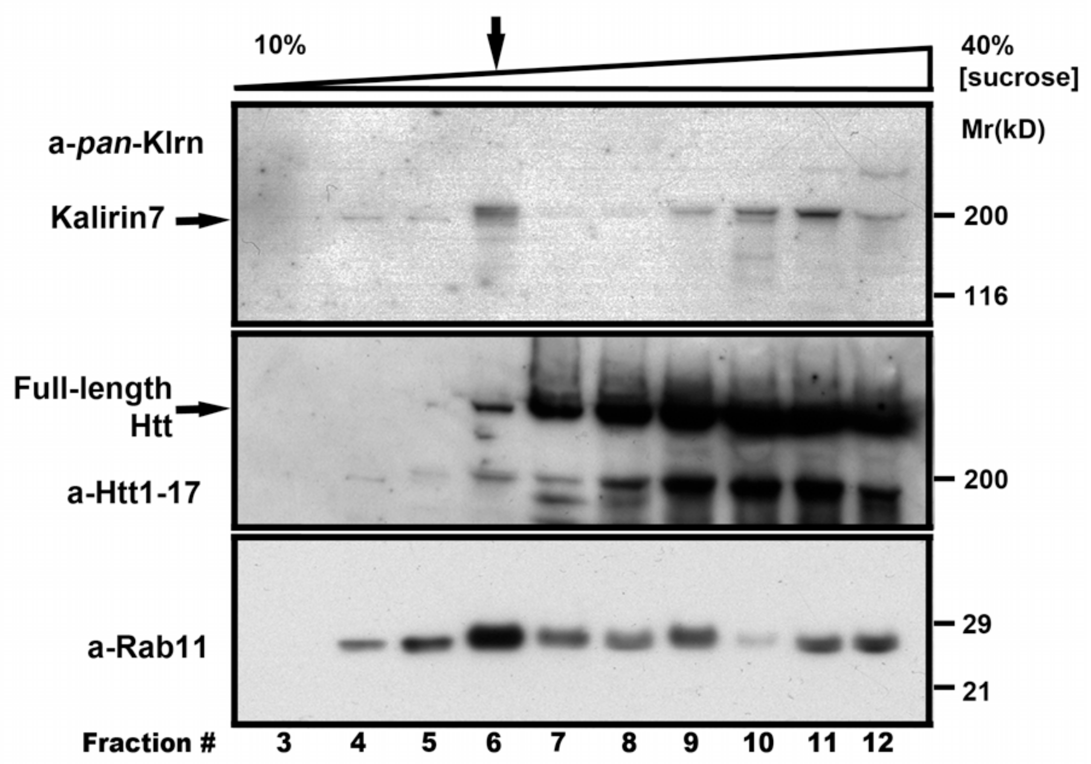

Supplementary figure S2. a, HAP1 was expressed at low levels in mouse embryonic cortex. Brain lysates of a WT adult mouse, WT mouse embryo and HAP1 KO mouse embryo were analyzed by Western blot with HAP1 antibodies purified with protein A/G. **b**, Precipitation of FLAG-tagged Htt fragments generated in **Figure 2a** from postnuclear supernatants of correspondingly transfected cells with anti-FLAG antibodies (clone-M2). After washed in lysis buffer, precipitated proteins on resins were eluted with 0.1M Tris/Cl (pH8,8), precipitated with chloroform/methanol, and resuspended in SDS-PAGE buffer for Western blot analysis with anti-FLAG antibodies (clone-M2).

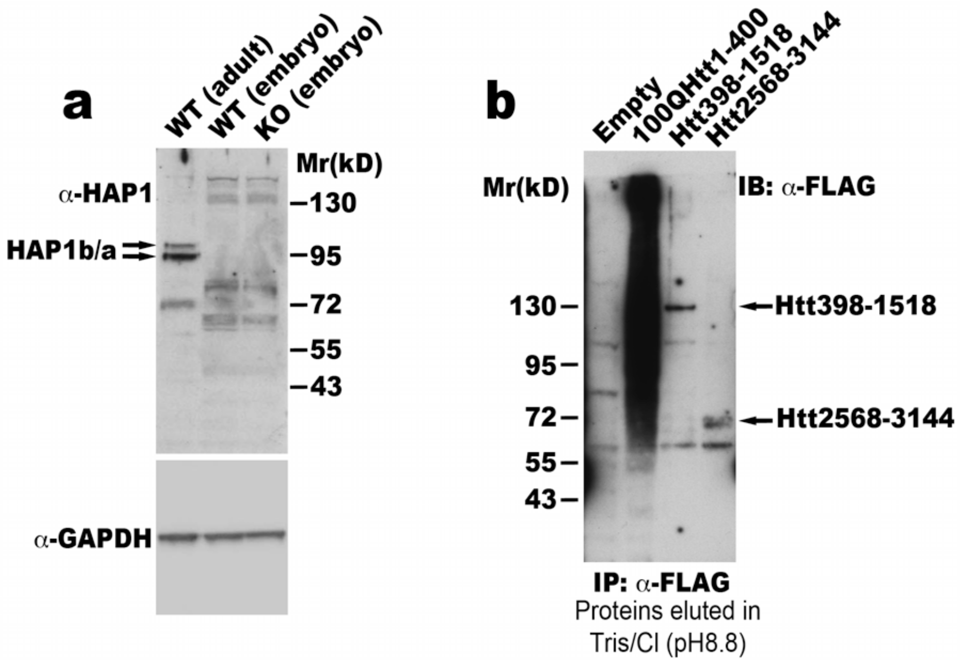

Supplementary figure S3. STHdh7Q/7Q striatal cells were transfected with plasmid DNAs expressing FLAG-tagged 18QHtt1-3144, 46QHtt1-3144, Klrn1-1654, Klrn23-684, Klrn674-1272, Klrn1269-1654, Rac1G12V or empty vector for 14 hours and processed for the MTT conversion assay. The same amount in molar mass for each plasmid was used for the transfection. Three independent transfections for each plasmid were conducted, and MTT conversion was measured in duplicate. Student t-test was carried out to compare the difference between the indicated condition and empty vector transfection (*, $p<0.05$).

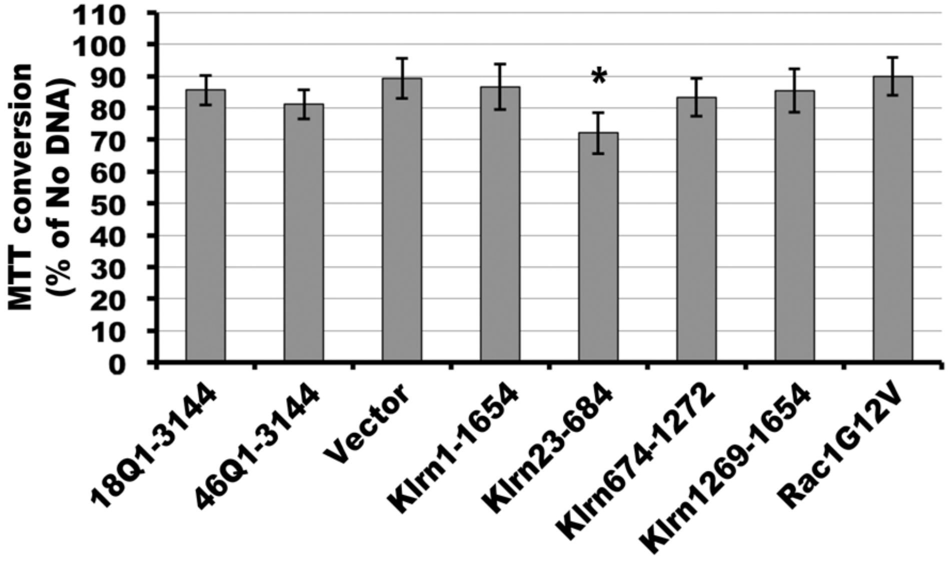

Supplementary whole blots/images for Figure 1a, 1b, 1c.

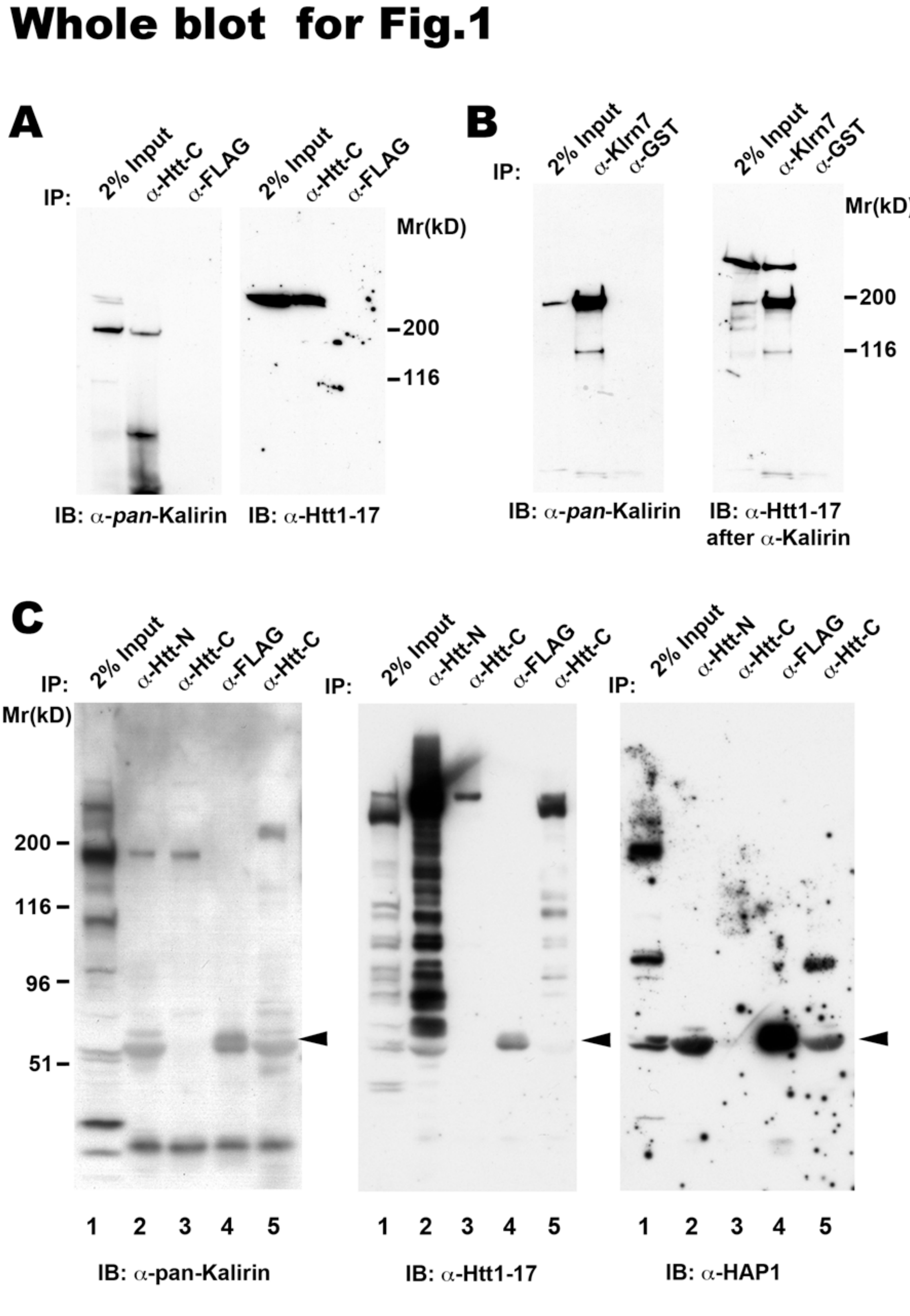

Supplementary whole blots/images for Figure 2b, 2d.

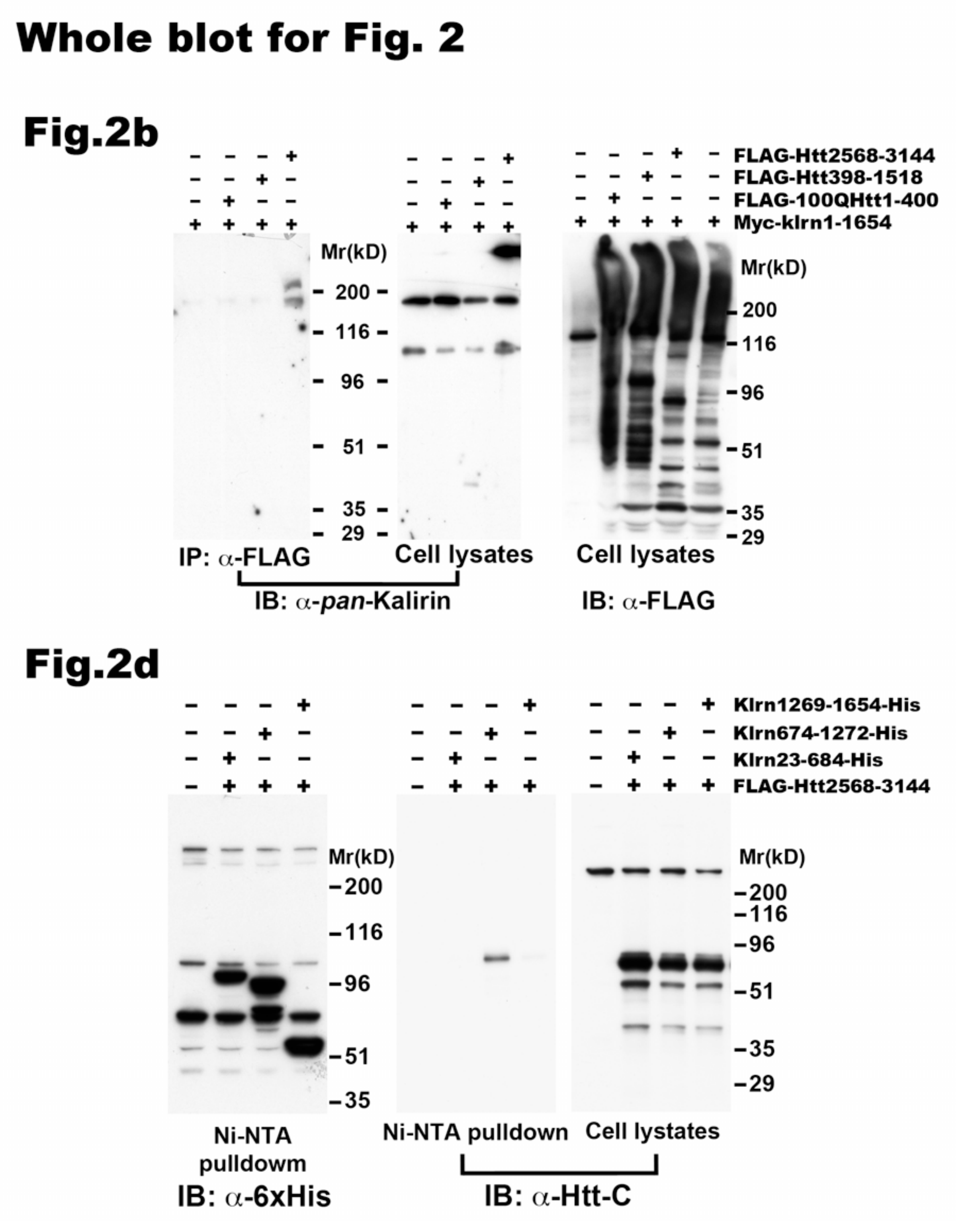

Reference:

1. McClory, H. *et al.* Glucose transporter 3 is a rab11-dependent trafficking cargo and its transport to the cell surface is reduced in neurons of CAG140 Huntington's disease mice. *Acta Neuropathol Commun* **2**, 179 (2014).
